# Impact of adding a limitations section to abstracts of systematic reviews on readers’ interpretation: a randomized controlled trial

**DOI:** 10.1186/1471-2288-14-123

**Published:** 2014-11-24

**Authors:** Amélie Yavchitz, Philippe Ravaud, Sally Hopewell, Gabriel Baron, Isabelle Boutron

**Affiliations:** Centre de Recherche Épidémiologies et Statistiques INSERM U1153, Paris, France; Centre of Clinical Epidemiology, Assistance Publique des Hôpitaux de Paris, Hôtel Dieu Hospital, Place du Parvis Notre-Dame, 75181 Paris, Cedex 4, France; Faculté de Médecine, French Cochrane Center, Paris Descartes University, Sorbonne Paris Cité, Paris, France; Department of Anesthesiology and Critical Care, Beaujon University Hospital, Clichy, France; Department of Epidemiology, Columbia University Mailman School of Public Health, New York, NY USA; Centre for Statistics in Medicine, University of Oxford, Wolfson College, Oxford, OX2 6UD UK

**Keywords:** Meta-analysis, Systematic review, Bias, Limits, Limitation, Interpretation, Interpretation bias, Misinterpretation, Abstract, Results

## Abstract

**Background:**

To allow an accurate evaluation of abstracts of systematic reviews, the PRISMA Statement recommends that the limitations of the evidence (e.g., risk of bias, publication bias, inconsistency, imprecision) should be described in the abstract. We aimed to evaluate the impact of adding such limitations sections on reader’s interpretation.

**Method:**

We performed a two-arm parallel group randomized controlled trial (RCT) using a sample of 30 abstracts of systematic reviews evaluating the effects of healthcare intervention with conclusions favoring the beneficial effect of the experimental treatments. Two formats of these abstracts were derived: one reported without and one with a standardized limitations section written according to the PRISMA statement for abstracts. The primary outcome was readers’ confidence in the results of the systematic review as stated in the abstract assessed by a Likert scale from 0, not at all confident, to 10, very confident. In total, 300 participants (corresponding authors of RCT reports indexed in PubMed) were randomized by a web-based randomization procedure to interpret one abstract with a limitations section (n = 150) or without a limitations section (n = 150). Participants were blinded to the study hypothesis.

**Results:**

Adding a limitations section did not modify readers’ interpretation of findings in terms of confidence in the results (mean difference [95% confidence interval] 0.19 [−0.37–0.74], p = 0.50), confidence in the validity of the conclusions (0.07 [−0.49–0.62], p = 0.80), or benefit of the experimental intervention (0.12 [−0.42–0.44], p = 0.65).

This study is limited because the participants were expert-readers and are not representative of all systematic review readers.

**Conclusion:**

Adding a limitations section to abstracts of systematic reviews did not affect readers’ interpretation of the abstract results. Other studies are needed to confirm the results and explore the impact of a limitations section on a less expert panel of participants.

**Trial registration:**

ClinicalTrial.gov (NCT01848782).

**Electronic supplementary material:**

The online version of this article (doi:10.1186/1471-2288-14-123) contains supplementary material, which is available to authorized users.

## Background

Systematic reviews are the cornerstone of therapeutic evaluation [1]. Clinicians, decision makers and researchers use them to keep up-to-date with current medical literature, develop clinical practice guidelines and sometimes plan future research [2–4]. However, systematic reviews may differ in quality depending on the conduct of the systematic review as well as the availability and quality of the primary studies [5]. Consequently, readers should carefully examine the methodological quality of reviews to assess their confidence in the results and conclusions.

Clinical decision makers and healthcare practitioners frequently rely on abstracts to decide the value of a study [6, 7]. In some cases, healthcare practitioners have access to only the abstract and make healthcare decisions based solely on the information in the abstract [8]. To improve the transparency of abstracts [9, 10], methodologists, researchers and editors have established recommendations for the presentation of systematic reviews in the PRISMA statements [11] with an extension for reporting abstracts [12]. These recommendations indicate that the limitations of the evidence should be described in the abstracts of systematic reviews [12]. This recommendation is rarely implemented [13], although some journals request that authors report a structured abstract with a limitations section [14]. However, the impact of adding this section to abstracts on readers’ interpretation is unknown.

We aimed to evaluate the impact of adding a limitations section, written according to the PRISMA statement for abstracts of systematic reviews, on readers’ interpretation of the abstract results.

## Methods

### Study design

We planned a randomized controlled trial to compare the interpretation of systematic review abstracts reported with and without a limitations section. We selected 30 abstracts of systematic reviews, and then developed 2 formats of the selected abstracts: reported with and without a limitations section written according to the PRISMA statement for abstracts. Then, we randomized participants to read and interpret one abstract with or without a limitations section. The study reporting follows the 2010 CONSORT statement [15].

### Selection of abstracts of systematic reviews

The abstracts were selected from a cohort of 100 systematic reviews assessing the effects of healthcare interventions, published between January and March 2012, and indexed in the Database of Reviews of Effects [13]. The search strategy and eligibility criteria for this cohort were described elsewhere [13]. From this cohort, we selected the first 30 systematic reviews whose abstract conclusion favored the beneficial effect of the experimental intervention and reported the risk of bias of primary studies in the full-text article, thus allowing selection of a relatively homogeneous sample. Abstracts from the cohort of 100 systematic reviews were screened by one author (AY) and reviewed by a second author (IB) to confirm eligibility. Any disagreements were resolved by consensus.

### Construction of the limitations section

According to the PRISMA statement for abstracts, the limitations section should address the following: 1) risk of bias common to many or all studies, such as lack of blinding for subjective outcomes or unavailability of data; 2) inconsistency of effect or association, as demonstrated by high heterogeneity; 3) imprecision due to few events or small sample size, for example; 4) indirectness of the evidence, such as use of an intermediate or short-term outcome; and 5) likelihood of publication bias [12, 16]. These limitations are the factors used to evaluate the level of evidence according to the GRADE approach [17].

One author (AY) systematically searched for and extracted from the full-text systematic review the following: 1) whether the systematic review had one or several of the limitations described above and 2) the limitations were reported by the authors of the systematic review in the full-text article or in the abstract. When limitations were outcome-specific, the limitations reported referred to the outcomes highlighted in the abstracts.

Then, for each selected abstract, one of the authors (AY) wrote a limitations section. The section focused on the limitations identified and was written in a standardized way, beginning with “This review is limited by….”, with a maximum of 2 sentences. When the original abstract was structured, we preserved the structured form and added the limitations section before the conclusions section with a heading “Limitations”. When the original abstract was not structured, we added the limitations section, without a heading, after the results and before the conclusion sentences.

We voluntarily did not use the same terminology as PRISMA because it may not be well understood by the readers (e.g., imprecision, inconsistency of effect or association and indirectness of the evidence are complex concepts). We kept the wording similar to that used by the authors of the systematic reviews, for example, when reporting the limitations in the discussion section. Another author (IB) read the entire modified abstract to determine whether the limitations section was written according to our specific guidance, that is, whether the limitations section 1) focused on the limitations previously identified in the report of the systematic review, 2) was no longer than 2 sentences, and 3) focused on limitations as described in the PRISMA for abstracts. When the second author found that the limitation section was not written according our specific guidance, the limitations section was re-written and discussed to reach consensus.

### Construction of abstracts with and without a limitations section

For each of the 30 selected abstracts, we obtained 1 abstract without a limitations section (i.e., the original abstract) and 1 with a limitations section (i.e., the original abstract plus the constructed limitations section). If the original abstract reported the limitations of the systematic review, this was deleted from the abstract without a limitations section and kept in the abstract with a limitations section. This situation occurred for 9 (30%) abstracts.

All abstracts were standardized, with the treatment name(s), authors and journal masked. The experimental treatment name was replaced by “intervention A”. When the control treatment was an active treatment, “comparator B” replaced the treatment name. Acronyms and abbreviations were also deleted.

An example of an abstract with and without a limitations section is given in the Table 1 and all abstracts with a limitations section are available in the Additional file 1.

**Table 1:** **Example of abstract construction**

Abstract without limitations section	Abstract with limitations section
**TITLE:** Comparative effectiveness of intervention A and comparator B for treatment of advanced urothelial carcinoma.	**TITLE:** Comparative effectiveness of intervention A and comparator B for treatment of advanced urothelial carcinoma.
**BACKGROUND:** Intervention A is a standard treatment of metastatic urothelial carcinoma (UC), though comparator B is frequently substituted due to improved tolerability. Because comparative effectiveness in clinical outcomes of intervention A - versus comparator B chemotherapy is lacking, a meta-analysis was carried out.	**BACKGROUND:** Intervention A is a standard treatment of metastatic urothelial carcinoma (UC), though comparator B is frequently substituted due to improved tolerability. Because comparative effectiveness in clinical outcomes of intervention A - versus comparator B chemotherapy is lacking, a meta-analysis was carried out.
**METHODS:** PubMed was searched for articles published from 1966 to 2010. Eligible studies included prospective randomized trials evaluating intervention A - versus comparator B regimens in patients with metastatic UC. Individual patient data were not available and survival data were inconsistently reported. Therefore, the analysis focused on overall response (OR) and complete response (CR) rates. The Mantel-Haenszel method was used for combining trials and calculating pooled risk ratios (RRs).	**METHODS:** PubMed was searched for articles published from 1966 to 2010. Eligible studies included prospective randomized trials evaluating intervention A - versus comparator B regimens in patients with metastatic UC. Individual patient data were not available and survival data were inconsistently reported. Therefore, the analysis focused on overall response (OR) and complete response (CR) rates. The Mantel-Haenszel method was used for combining trials and calculating pooled risk ratios (RRs).
**RESULTS:** A total of 286 patients with metastatic UC from four randomized trials were included. Intervention A was associated with a significantly higher likelihood of achieving a CR [RR =3.54; 95% confidence interval (CI) 1.48-8.49; P =0.005] and OR (RR =1.34; 95% CI 1.04-1.71; P =0.02). Survival end points could not be adequately assessed due to inconsistent reporting among trials.	**RESULTS:** A total of 286 patients with metastatic UC from four randomized trials were included. Intervention A was associated with a significantly higher likelihood of achieving a CR [RR =3.54; 95% confidence interval (CI) 1.48-8.49; P =0.005] and OR (RR =1.34; 95% CI 1.04-1.71; P =0.02). Survival end points could not be adequately assessed due to inconsistent reporting among trials.
**CONCLUSIONS:** Intervention A, as compared with comparator B, significantly increases the likelihood of both OR and CR in patients with metastatic UC. The impact of improved response proportions on survival end points could not be assessed.	**LIMITATIONS:** This review is limited by the small sample sizes and methodological quality of the included studies. None of the included studies was blinded or placebo controlled; two studies closed early.
	**Conclusions:** Intervention A, as compared with comparator B, significantly increases the likelihood of both OR and CR in patients with metastatic UC. The impact of improved response proportions on survival end points could not be assessed.

### Participants

Eligible participants were the corresponding authors of clinical trials published between January 2010 and June 2013 and indexed in PubMed Core Clinical Journals, with an email address available on PubMed.

Potential participants were invited by e-mail to participate in an online survey on the interpretation of abstracts of systematic reviews. As an incentive, they were told that they would be entered into a draw along with all other participants for a chance to win an Apple iPad mini. If they agreed to participate, they logged onto a secure website and answered a screening question asking if they were a clinician; non-clinicians were excluded. Invitation emails were sent in waves until the planned number of clinicians had logged on and completed the assessment. A maximum of 2 reminders were sent to participants. The email invitation and details of the questionnaire are in the Additional file 1.

### Randomization

A computer-generated randomization list was generated for allocating participants to abstracts with or without a limitations section in a 1:1 ratio. Allocation was concealed by use of a web-based randomization procedure whereby participants logged onto the system and were randomized to evaluate an abstract with or without a limitations section. Clinicians who logged onto the system but did not evaluate an abstract were excluded and the abstract was automatically allocated to another clinician.

### Blinding

Participants were blinded to the study’s hypothesis. They were informed that they were participating in a study on the interpretation of abstracts of systematic reviews, but they were not aware that they were randomized to assess an abstract with or without a limitations section.

### Outcomes measures

The primary outcome was readers’ confidence in the results of the systematic review as stated in the abstract (i.e., *How confident are you in the results of the systematic review?)* assessed on a Likert scale, from 0 “not at all confident” to 10 “very confident”. The secondary outcomes were the confidence in the validity of the conclusions (i.e., *How confident are you in the validity of the conclusion of this study?)*, the beneficial effect of the experimental intervention (i.e., *How confident are you that intervention “A” could be of benefit to patients?)*, the influence of the results on clinical practice (i.e., *How confident are you that the results of this study could influence your clinical practice?)* and the rigor of the systematic review (i.e., *Do you think that this systematic review was conducted rigorously?*) assessed on an 11-point Likert scale.

### Sample size

Each participant read 1 abstract with or 1 abstract without a limitations section. The unit of analysis was the abstract. A sample of 266 participants was theoretically needed to be able to detect an effect size of 0.4 with the primary outcome (with power of 90% and alpha risk 5%). An effect size of 0.4 is equivalent to a decrease in the primary outcome of 1 point (the minimum expected difference between groups on a 0–10 scale) with an SD of 2.5. Theoretically, each abstract must be read the same number of times according to the randomization group. Knowing that each participant would read only one abstract (with or without a limitations section), we chose to include 300 participants. Each abstract with and without a limitations section was assessed 5 times.

### Statistical analysis

Statistical analysis involved use of SAS v9.3 (SAS Inc., Cary, NC). All outcomes were quantitative; differences between groups were analyzed by a linear mixed model with a fixed factor (group) and random abstracts and abstract × group interaction effects. A random-effects model allowed for taking into account 2 levels of clustering: by abstract (each abstract was assessed 5 times in each group) and interclustering (pairing between the abstracts used in the 2 arms of the trial). Inference was based on restricted maximum likelihood. For primary outcome and secondary outcomes, we estimated the difference between means (with 95% confidence intervals [95% CIs]) for abstracts with and without a limitations section. P <0.05 was considered statistically significant.

### Ethical considerations

The institutional review board from the University of Paris Descartes approved the protocol No. CL178200001. The study is registered in ClinicalTrials.gov (no. NCT01848782).

## Results

### Characteristics of participants

**Figure 1:**
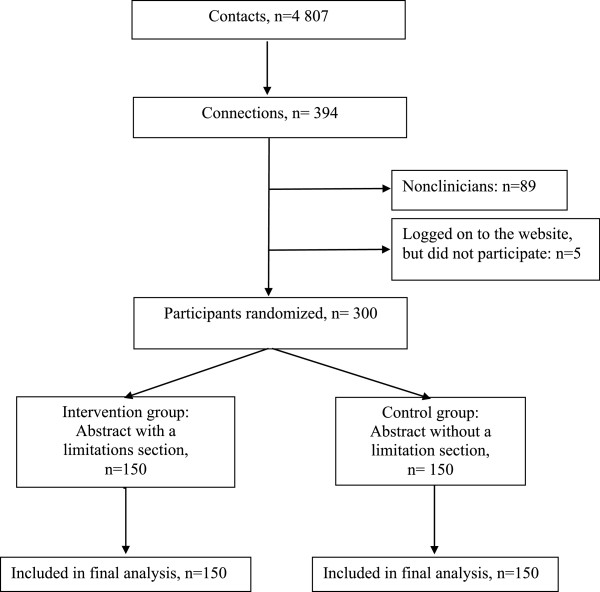
**Flow chart of participants in the trial.**

Among the 4,807 potential participants who were invited by e-mail to participate in the survey between May 1 and June 30, 2013, 394 logged onto the study site; 89 were excluded because they were not clinicians and 5 did not complete the survey. From the 300 participants, 150 were randomized to the intervention group (i.e., abstracts with a limitations section), and 150 to the control group (i.e., abstracts without a limitations section). In total, 150 participants per group were included in the final analysis (Figure 1).

The median [Q1-Q3] participant age was 45 [range 38–54] years; 72% were male. Participants were mainly located in the European Union (49%) and in the United States (33%). Most had medical experience (74.7% had been clinicians for more than 10 years) and more than half (59%) regularly read reports of systematic reviews. More than half (53%) had been involved in a systematic review, but 46% had never peer-reviewed a systematic review for a biomedical journal (Table 2).

**Table 2:** **Characteristics of participants**

		Abstract without limitations n (%)	Abstract with limitations n (%)	Total n (%)
		N = 150	N = 150	N = 300
**Qualification**	MD	78 (52.0)	78 (52.0)	156 (52.0)
	PhD	58 (38.7)	64 (42.7)	122 (40.7)
	Other	14 (9.3)	8 (5.3)	22 (7.3)
**Clinical experience**	<5 years	6 (4.0)	8 (5.3)	14 (4.7)
	5 to 10 years	34 (22.7)	28 (18.7)	62 (20.7)
	>10 years	110 (73.3)	114 (76.6)	224 (74.7)
**Reading reports of systematic reviews**	Rarely or sometimes	61 (40.7)	62 (41.6)	123 (41.1)
	Regularly	89 (59.3)	87 (58.4)	176 (58.9)
**No. of randomized trials involved in**	0	16 (10.7)	11 (7.3)	27 (9.0)
	1–3	48 (32.0)	48 (32.0)	96 (32.0)
	4–9	45 (30)	54 (36.0)	99 (33.0)
	>10	41 (27.3)	37 (24.7)	78 (26.0)
**Authored at least one systematic review**		77 (51.3)	82 (55.0)	159 (53.2)
**No. of systematic reviews peer-reviewed**	0	75 (50.0)	64 (43.0)	139 (46.5)
	1–3	55 (36.7)	69 (46.3)	124 (41.5)
	>3	20 (13.3)	16 (10.7)	36 (12.0)
**Training in clinical epidemiology**		101 (67.3)	76 (50.7)	177 (59.0)
**Training in methods of randomized trials**		86 (57.3)	71 (47.3)	157 (52.3)

### Characteristics of systematic reviews, abstracts and limitations sections

The general characteristics of the included systematic reviews and the quality of the reporting of the abstracts (according to the PRSIMA statement for abstracts [12]) are in Table 3.

**Table 3:** **Characteristics of systematic reviews included**

Characteristics		N = 30
**Field of research, n (%)**	Medicine	27 (90)
	Surgery	3 (10)
**Impact factor, median; [Q1-Q3]; (min-max)**		4.60; [3.18–7.51]; (0.00-18.04)
**No. of trials included, median; [Q1-Q3]; (min-max)**		13; [6–23]; (4–54)
**Sample size, median; [Q1-Q3]; (min-max)**		2872; [801–11005]; (257–782460)
**Experimental treatment, n (%)**	Drugs	16 (53)
	Complex intervention	11 (37)
	Surgery	2 (7)
	Device	1 (3)
**Meta-analysis included in the systematic review, n (%)**		26 (87)
**Funding source, n (%)**	Non-profit	11 (37)
	For-profit	2 (7)
	None	10 (33)
	Not specified	7 ((23)
**Content of the original abstract, n (%)**	Eligibility criteria	17 (57%)
	Key data bases and date of search	9 (30%)
	Method to assess the risk of bias	6 (20%)
	No. and type of the studies included in the systematic review	22 (73%)
	Summary measure and confidence interval for the main outcome results*	20/26 (77%)
	Strength and limitation	13 (43%)

*This result applies on the 26 systematic reviews that included a meta-analysis.

Overall, 23 abstracts were structured abstracts, and we created a specific heading for the limitations section, whereas 7 abstracts were not structured, and a sentence reporting the limitations was added before the conclusions. Some limitations were reported in 9 original abstracts. These limitations were deleted and rewritten according to the PRISMA guidelines. The quality assessment of the risk of bias in the selected systematic reviews was assessed with different tools. Authors of the included systematic reviews used the Cochrane or a modified Cochrane Collaboration tool in 10 abstracts (33%), the Jadad scale or modified Jadad scale in 5 (17%), the PEDro scale or modified PEDro scale in 3 (10%), and other tools in 4 (13%); the scale was not specified in 6 (20%).

The limitations sections we created focused on risk of bias in 22 abstracts (73%), heterogeneity in 13 (43%), publication bias in 15 (50%), imprecision of results in 12 (40%), and indirectness of the evidence in 2 (7%) abstracts. The median [Q1-Q3] word count for limitations sections was 27 [23–31]. The median number of limits described in the limitations sections was 2 (range 1–4).

### Clinicians’ interpretation of abstracts

Readers’ assessment of abstracts with and without a limitations section did not differ in the primary outcome -- confidence in the results (mean [SD] =4.4 [2.3] and 4.6 [2.5], respectively; mean difference [95% CI] 0.2 [−0.4 to 0.7], p = 0.5). For the secondary outcomes, the assessment of abstracts with and without a limitations section did not differ for confidence in the validity of the conclusions (mean difference 0.07 [−0.5 to 0.6], p = 0.8); benefit of the experimental intervention to patients (mean difference 0.1 [−0.4 to 0.7], p = 0.6); influence of the results on clinical practice (mean difference −0.08 [−0.6 to 0.5], p = 0.8) and rigor of the systematic review (mean difference −0.4 [−1.0 to 0.2], p = 0.2) (Table 4).

**Table 4:** **Results of primary and secondary outcomes**

	Abstract without limitations	With Limitations	Mean difference (95% CI)	p-value
	Mean (SD) n = 150	Mean (SD) n = 150		
**How confident are you in the results of this study (0–10)?**	4.6 (2.5)	4.4 (2.3)	0.2 (−0.4 to 0.7)	p =0.5
**How confident are you in the validity of the conclusion of this study (0–10)?**	4.1 (2.5)	4.0 (2.3)	0.07 (−0.5 to 0.6)	p =0.8
**How confident are you that the intervention A could be benefit to patients (0–10)?**	4.4 (2.6)	4.3 (2.3)	0.1 (−0.4 to 0.7)	p =0.6
**How confident are you that the results of this study could influence your clinical practice (0–10)?**	3.8 (2.6)	3.8 (2.3)	−0.08 (−0.6 to 0.5)	p =0.8
**Do you think that this systematic review was conducted rigorously (0–10)?**	4.1 (2.7)	4.4 (2.6)	−0.4 (−1.0 to 0.2)	p =0.2

95% CI, 95% confidence interval.

## Discussion

This study evaluated, in a randomized controlled trial, the impact of adding a limitations section to an abstract on the interpretation of abstracts of systematic reviews. This randomized controlled trial involved a large international sample of clinicians and a sample of “real life” abstracts of systematic reviews (i.e., abstracts of published systematic reviews indexed in DARE). Despite the selection of a sample of systematic reviews of good quality, the mean confidence of readers was low, and adding a limitations section had no impact on the interpretation of abstract results by expert-readers.

Because abstracts are the first and sometimes the only source of information for readers, editors are attentive to their quality and their capacity to provide all the necessary and important information on the research performed. In the 1960s, abstracts were usually reported on the last page of research articles and were moved to the beginning of research articles [18]. Since then, many editorial policies have been implemented to try to improve the content and the format of abstracts. These policies have involved the development and implementation of structured abstracts [18, 19], reporting guidelines such as the CONSORT for abstracts of randomized controlled trials and the PRISMA statement for abstracts of systematic reviews [12, 20]. Such policies can improve the quality of reporting of abstracts [21] and should in theory improve the interpretation by readers.

However, despite these initiatives, the quality of reporting of abstracts remains questionable [9, 10, 22–24]. A recent study showed that despite systematic reviews including primary studies with high risk of bias, just over half included a risk of bias assessment in the interpretation of results in the abstract [13]. Consequently, adding a limitations section could be useful to enhance readers’ awareness and improve their interpretation. However, a limitations section in the abstract is recommended by only a few journals and for systematic reviews in the PRISMA statement for abstracts [12]. For example, *Annals of Internal Medicine* has required authors to include a limitations section in the abstract of scientific articles since 2004 [14].

To our knowledge, the impact of adding a limitations section in abstracts of systematic reviews has never been evaluated. Previous studies have evaluated the impact of different reporting on the interpretation of the study by readers. These studies mainly involved use of a single abstract of a fictional trial. For example, industry sponsorship can negatively influence the perception of the methodological quality of a study and the willingness to believe the study findings [25]. Similarly, interpretation of study results is affected by the reporting of outcomes as absolute risk, relative risk or number needed to treat [26, 27].

Our results did not show any impact of the abstract limitations section on expert-readers’ interpretation. Furthermore, our results highlight that confidence in results was low in both arms. The high level of expertise of the participants could explain these results. In fact, half of the clinicians included in this randomized controlled trial had some experience in the conduct and reviewing of systematic reviews. This level of expertise could increase their awareness of the common limitations of systematic reviews such as the risk of publication bias or the limited quality of primary studies. Furthermore, the limitations section reported factual information and in a neutral form, and the conclusion of the systematic reviews’ abstract was not modified. Also, assessing the confidence in the results of a systematic review is complex because it depends both on how the systematic review is conducted and the quality of the primary studies included.

Our study has some limitations. First, the readers did not access the full-text article to fully appraise the study results; they only assessed an abstract with or without a limitations section. However abstracts of systematic reviews are very important, because some readers cannot access full-text articles because of the fee requirement, low Internet download capacity, or the full-text article being available only in a language not understood by the reader. Second, the participants were corresponding authors of articles of randomized trials and systematic reviews, who may be considered “reader-experts” and not representative of all readers of systematic reviews. Consequently, we cannot exclude that a limitations section may be useful for a less expert readership. Finally, we explored only the impact of a limitations section added to abstracts reporting a systematic review and we cannot extrapolate our findings to limitations sections in abstracts reporting other types of studies such as randomized controlled trials and observational studies.

However, this study has important implications. At this stage, we cannot make any recommendations for practice and we should probably not change guidelines and editorial policies related to the reporting of a limitations section in abstracts of systematic reviews. However, this study highlights an important topic for future research. First, because our study is the first study on this topic, the trial should be replicated, and other trials including a less expert readership or with different background (e.g. authors of “clinical practice guidelines”) should be performed. Second, qualitative studies would probably be useful to help define how limitations sections should be reported to have a real impact on readers. Third, we recommend exploring the impact of a limitations section in abstracts of other study designs such as randomized controlled trials and observational studies. Overall, more research is needed on the interpretation of research results from abstracts because abstracts are widely disseminated.

## Conclusions

In conclusion, adding a limitations section in abstracts of systematic review may not affect expert-readers’ interpretation of abstract results and conclusions. Future studies are needed to confirm these results and explore the impact of a limitations section on a less expert panel of participants.

## Authors’ information

On-line survey design and data management: Isabelle Pane, senior computer engineer and data manager, and Joan Denis, junior computer engineer and data manager, Centre de recherche Épidémiologies et Biostatistiques, INSERM U1153, Paris, France.

## Electronic supplementary material

Additional file 1: **The 30 Abstracts with limitations section. The invitation e-mail for the participants. The survey.** (DOCX 56 KB)
