# Supplementary material for: Impact of adding a limitations section to abstracts of systematic reviews on readers’ interpretation: a randomized controlled trial
Source: BMC Med Res Methodol. 2014 Nov 24;14:123. doi: 10.1186/1471-2288-14-123 (PMC4247631; doi:10.1186/1471-2288-14-123)
Supplement: Supplementary file 1 — Additional file 1: The 30 Abstracts with limitations section. The invitation e-mail for the participants. The survey. (DOCX 56 KB) [file 12874_2014_1130_MOESM1_ESM.docx]

### Appendix

Abstracts with limitations section highlight in red

## Abstract 1:

### Titre:

Impact of intervention A on sustained virological response to peginterferon and ribavirin therapy for HCV infection: a systematic review and meta-analysis.

### Abstract:

Anaemia is a common complication of antiviral therapy for chronic hepatitis C virus (HCV) infection that necessitates dose reductions or therapy discontinuation. Administration of intervention A is an alternative to ribavirin (RBV) dose reduction, but its advantage in terms of sustained virological response (SVR) has not been determined yet. In a systematic way, randomized studies were identified that evaluated the effect of intervention A administration vs RBV dose reduction on virological response in patients who developed anaemia during anti-HCV therapy. The random-effects model was employed to run meta-analysis. SVR was set as the end point of interest. Data were abstracted from four studies containing 257 patients who developed anaemia during therapy. One hundred and twenty six subjects underwent RBV dose reduction. Patients who received intervention A in response to haemoglobin drop had a significantly higher probability of achieving SVR compared with those who underwent RBV dose reduction because of anaemia (relative risk = 1.83 95% CI; 1.41-2.37). No heterogeneity was observed across study results (I2 = 0). Publication bias assessment was nonsignificant. This review is limited by the low methodological quality of the included studies and the limited number of studies making publication bias assessment problematic. Our meta-analysis indicates that administration of intervention A in patients who develop anaemia during anti-HCV therapy can considerably enhance SVR. Moreover, no adverse event of intervention A administration was reported among included subjects.

## Abstract 2

### Titre:

How effective were interventions A in real-world settings that were modeled on the Diabetes Prevention Program?

### Abstract:

We conducted a systematic review and meta-analysis of twenty-eight US-based studies applying the findings of the Diabetes Prevention Program, a clinical trial that tested the effects of intervention A for people at high risk for diabetes, in real-world settings. The average weight change at twelve months after the intervention was a loss of about 4 percent from participants' baseline weight. Change in weight was similar regardless of whether the intervention was delivered by clinically trained professionals or lay educators. Additional analyses limited to seventeen studies with a nine-month or greater follow-up assessment showed similar weight change. With every additional intervention A session attended, weight loss increased by 0.26 percentage point. This review is limited by the heterogeneity in study design (among the 28 studies included, four were randomized trials, two cluster trials, twenty single group pre-post studies, and two nonrandomized controlled studies), the methodological quality of the included studies and the possible publication bias. We conclude that costs associated with diabetes prevention can be lowered without sacrificing effectiveness, using nonmedical personnel and motivating higher attendance at program sessions.

## Abstract 3

### Titre:

Meta-analysis: intervention A improves liver histology and fibrosis in patients with non-alcoholic steatohepatitis.

### Background:

Intervention A has been used in the treatment of non-alcoholic steatohepatitis (NASH). However, the magnitude of treatment response associated with intervention A in improving liver histology in NASH has not been quantified systematically.

### Aim:

To conduct a meta-analysis of randomised, placebo-controlled clinical trials (RPCTs) using intervention A in the treatment of NASH.

### Methods:

Pubmed/MEDLINE and Cochrane Central Register of Controlled Trials 2010 were searched until September 2010 and four RPCTs were identified. Peto odds ratios (ORs) and their respective 95% confidence intervals (CIs) were used to assess the efficacy of intervention A in improving liver histological parameters.

### Results:

Four good quality RPCTs derived from three continents were included. The meta-analysis showed that intervention A (n = 169) was significantly better than placebo (n = 165) in improving ballooning degeneration, lobular inflammation and steatosis with combined ORs of 2.11 (95% CI, 1.33-3.36), 2.58 (95% CI, 1.68-3.97) and 3.39 (95% CI, 2.19-5.25) respectively. The improvement in combined necroinflammation with intervention A (n = 58) vs. placebo (n = 52) was also statistically significant (combined OR 6.52[95% CI, 3.03-14.06]), but improvement in fibrosis was not. When intervention A (n = 137) was analysed alone, the improvement in fibrosis with intervention A (n = 137) vs. placebo (n = 134) (combined OR 1.68 [95% CI, 1.02-2.77]) was statistically significant. The total body fat slightly decreased in the control, while it markedly and highly significantly increased with intervention A.

### Limitations:

This review is limited by the small sample size of the trials included. Further, because of the small number of trials included, publication bias cannot be excluded.

### Conclusions:

Intervention A significantly improves ballooning degeneration, lobular inflammation, steatosis and combined necroinflammation in patients with NASH. Intervention A may improve fibrosis. Larger randomised, placebo-controlled clinical trials are needed to examine the efficacy of intervention A in improving NASH fibrosis.

## Abstract 4

### Titre:

Quality of life and related dimensions in cancer patients treated with intervention A: a meta-analysis.

### Objectives:

The aim of this meta-analysis was to determine the effectiveness of the intervention A, in the treatment of patients with cancer with respect to quality-of-life- (QoL-) associated measures.

### Methods:

We searched databases such as PubMed/Medline, Excerpta Medica Database (EMBASE), CAMbase, and other for controlled clinical studies on parameters associated with QoL. Outcome data were extracted and converted into standardized mean differences and their standard errors.

### Results:

Thirteen prospective and controlled studies which met the inclusion/exclusion criteria reported positive effects in favor of the intervention A. A random-effect meta-analysis estimated the overall treatment effect at standardized mean difference = 0.56 (CI: 0.41 to 0.71, P < .0001).

### Limitations:

This systematic review is limited by the very poor methodological quality of the included studies. Further, there is some evidence of publication bias.

### Conclusions:

The analyzed studies give some evidence that intervention A might have beneficial short-time effects on QoL-associated dimensions and psychosomatic self-regulation.

## Abstract 5

### Titre:

Impact of intervention A during pregnancy on atopic eczema in childhood--a meta-analysis.

### Abstract:

In the present study, we sought to conduct a literature review of randomised, double-blind, placebo-controlled trials, which assessed the impact of intervention A on the development of eczema in children. A meta-analysis was conducted for comparison of the development of atopic eczema in children whose mothers tooks intervention A v. placebo. Study selection, quality appraisal and data extraction were performed independently and in duplicate. The studies were rated according to their size in order to calculate the influence of individual studies on the meta-analysis. A total of seven randomised, double-blind, placebo-controlled trials, published between 2001 and 2009, were selected from the PubMed and Ovid databases for the meta-analysis. The meta-analysis was performed with statistical software Stata/SE11.0. The completed meta-analysis of the seven studies shows a significant risk reduction for atopic eczema in children aged 2-7 years by the administration of intervention A during pregnancy (reduction 5.7 %; P = 0.022). However, this effect was only significant for intervention A type 1 (reduction 10.6 %; P = 0.045), but not for intervention A type 2 (difference 3.06 %, P = 0.204). This review is limited by the lack of precision; the effect can be ascribed only to the results of three of the seven included studies. In conclusion, the meta-analysis shows that the administration of intervention A type 1 during pregnancy prevents atopic eczema in children aged from 2 to 7 years. However, intervention A type 2 does not affect the development of atopic eczema, independent of whether they contain intervention A type 1 or not.

## Abstract 6

### Titre:

Intervention A in the management of aneurismal subarachnoid hemorrhage revisited. A meta-analysis

### Background:

To reassess the use of intervention A in the management of aneurysmal subarachnoid hemorrhage (SAH) in the setting of present-day treatment strategies.

### Methods:

The authors conducted a systematic review of the literature and a meta-analysis. They reviewed the PubMed database and conducted a manual review of article bibliographies.

### Results:

Using a pre-specified search strategy, 17 relevant studies involving a total of 2,872 patients with SAH at baseline, from which data of 1,380 patients having received intervention A, were included in a meta-analysis. Pooled odds ratios of the impact of intervention A on functional outcomes, rebleeding, and cerebral infarction were calculated. Short-term use of intervention A (72 h or less) associated with medical prevention of ischemic deficit seems to yield better results on functional outcome than long-term use of intervention A, especially if not associated with a medical prevention of ischemic deficit. The risk of cerebral infarction is not increased by the shortterm use of intervention A and the risk of rebleeding is decreased independently of the length of intervention A use.

### Limitations:

This review is limited by the methodological quality of the included studies (five studies were observational studies). Further we cannot exclude the possibility of publication bias.

### Conclusions:

The use of intervention A should be reconsidered in the setting of modern-era treatment strategies, as the short-term use associated with medical prevention of ischemic deficit decreases the rate of rebleeding and does not increase the risk of cerebral infarction, thus potentially yielding better protection against poor functional outcome.

## Abstract 7

### Titre:

Comparative effectiveness of intervention A and comparator B for treatment of advanced urothelial carcinoma.

### Background:

Intervention A is a standard treatment of metastatic urothelial carcinoma (UC), though comparator B is frequently substituted due to improved tolerability. Because comparative effectiveness in clinical outcomes of intervention A - versus comparator B chemotherapy is lacking, a meta-analysis was carried out.

### Methods:

PubMed was searched for articles published from 1966 to 2010. Eligible studies included prospective randomized trials evaluating intervention A - versus comparator B regimens in patients with metastatic UC. Individual patient data were not available and survival data were inconsistently reported. Therefore, the analysis focused on overall response (OR) and complete response (CR) rates. The Mantel-Haenszel method was used for combining trials and calculating pooled risk ratios (RRs).

### Results:

A total of 286 patients with metastatic UC from four randomized trials were included. Intervention A was associated with a significantly higher likelihood of achieving a CR [RR = 3.54; 95% confidence interval (CI) 1.48-8.49; P = 0.005] and OR (RR = 1.34; 95% CI 1.04-1.71; P = 0.02). Survival end points could not be adequately assessed due to inconsistent reporting among trials.

### Limitations:

This review is limited by the small sample sizes and methodological quality of the included studies. None of the included studies were blinded or placebo controlled, two studies closed early.

### Conclusions:

Intervention A, as compared with comparator B, significantly increases the likelihood of both OR and CR in patients with metastatic UC. The impact of improved response proportions on survival end points could not be assessed.

## Abstract 8

### Titre:

Meta-analysis of intervention A to reduce pain in patients with cancer.

### Purpose:

Pain is one of the most common, burdensome, and feared symptoms experienced by patients with cancer. American Pain Society standards for pain management in cancer recommend both pharmacologic and psychosocial approaches. To obtain a current, stable, and comprehensive estimate of the effect of intervention A on pain-an important clinical topic-we conducted a meta-analysis of randomized controlled studies among adult patients with cancer published between 1966 and 2010.

### Methods:

Three pairs of raters independently reviewed 1,681 abstracts, with a systematic process for reconciling disagreement, yielding 42 papers, of which 37 had sufficient data for meta-analysis. Studies were assessed for quality using a modified seven-item Physiotherapy Evidence Database (PEDro) coding scheme. Pain severity and interference were primary outcome measures.

### Results:

Study participants (N = 4,199) were primarily women (66%) and white (72%). The weighted averaged effect size across studies for pain severity (38 comparisons) was 0.34 (95% CI, 0.23 to 0.46; P < .001), and the effect size for pain interference (four comparisons) was 0.40 (95% CI, 0.21 to 0.60; P < .001). Studies that monitored whether treatment was delivered as intended had larger effects than those that did not (P = .04).

### Limitations:

This review is limited by the methodological quality of the included studies (fewer than 20% of studies concealed allocation or blinded assessors, and fewer than half of all studies reported monitoring treatment implementation). Also not all studies measured pain as the primary outcome.

### Conclusions:

Intervention A had medium-size effects on both pain severity and interference. These robust findings support the systematic implementation of quality-controlled interventions A as part of a multimodal approach to the management of pain in patients with cancer.

## Abstract 9

### Titre:

Intervention A effects on depressive symptoms in cancer survivors: a systematic review and meta-analysis.

### Background:

Depression is a distressing side effect of cancer and its treatment. In the general population, intervention A is an effective antidepressant.

### Objectives:

We conducted a systematic review and meta-analysis to determine the antidepressant effect of intervention A in cancer survivors.

### Data sources:

In May 2011, we searched MEDLINE, PsycInfo, EMBASE, CINAHL, CDSR, CENTRAL, AMED, Biosis Previews, and Sport Discus and citations from relevant articles and reviews.

### Study eligibility criteria:

We included randomized controlled trials (RCT) comparing intervention A with usual care in cancer survivors, using a self-report inventory or clinician rating to assess depressive symptoms, and reporting symptoms pre- and postintervention.

### Study appraisal:

Around 7,042 study titles were identified and screened, with 15 RCTs included.

### Synthesis methods:

Effect sizes (ES) were reported as mean change scores. The Q test was conducted to evaluate heterogeneity of ES. Potential moderator variables were evaluated with examination of scatter plots and Wilcoxon rank-sum or Kruskal-Wallis tests.

### Results:

The overall ES, under a random-effects model, was -0.22 (confidence interval, -0.43 to -0.09; P = 0.04). Significant moderating variables (ps < 0.05) were intervention A location, intervention A supervision, and intervention A duration.

### Limitations:

Small sample sizes of the included studies, heterogeneity of results and evidence of publication bias limit this review. Only one study identified depression as the primary endpoint and only two studies reported a blinded outcome assessment.

**Conclusions:** Intervention A has modest positive effects on depressive symptoms with larger effects for programs that were supervised or partially supervised, not conducted at home, and at least 30 minutes in duration.

### Impact:

Our results complement other studies showing that intervention A is associated with reduced pain and fatigue and with improvements in quality of life among cancer survivors.

## Abstract 10

### Titre:

Body composition and quality of life in adults treated with intervention A: a systematic review and meta-analysis.

### Objectives:

To summarize the evidence about the efficacy and safety of using intervention A in adults with GH deficiency focusing on quality of life and body composition.

### Data sources:

We searched MEDLINE, EMBASE, Cochrane CENTRAL, Web of Science and Scopus through April 2011. We also reviewed reference lists and contacted experts to identify candidate studies.

### Study selection:

Reviewers, working independently and in duplicate, selected randomised controlled trials (RCTs) that compared intervention A to placebo.

### Data synthesis:

We pooled the relative risk (RR) and weighted mean difference (WMD) by the random effects model and assessed heterogeneity using the I(2) statistic.

### Results:

Fifty-four RCTs were included enrolling over 3400 patients. Intervention A use was associated with statistically significant reduction in weight (WMD, 95% confidence interval (95% CI): -2.31kg, -2.66 and -1.96) and body fat content (WMD, 95% CI: -2.56kg, -2.97 and -2.16); increase in lean body mass (WMD, 95% CI: 1.38, 1.10 and 1.65), the risk of oedema (RR, 95% CI: 6.07, 4.34 and 8.48) and joint stiffness (RR, 95% CI: 4.17, 1.4 and 12.38); without significant changes in body mass index, bone mineral density or other adverse effects. Quality of life measures improved in 11 of the 16 trials although meta-analysis was not feasible.

### Limitations:

This review is limited by the heterogeneity in outcome reporting and the lack of quantitative data reported for quality of life.

### Conclusions:

Intervention A therapy in adults with confirmed GH deficiency reduces weight and body fat, increases lean body mass and increases oedema and joint stiffness. Most trials demonstrated improvement in quality of life measures.

## Abstract 11

### Titre:

Universal voluntary HIV testing in antenatal care settings: a review of the intervention A.

### Objectives:

To assess the contribution of intervention A to achieving universal testing of pregnant women and, from available data on components of intervention A, assess whether intervention A adoption adheres to pre-test information, post-test counselling procedures and linkage to treatment.

### Methods:

Systematic review of published literature. Findings were collated and data extracted on HIV testing uptake before and after the adoption of an intervention A model. Data on pre- and post-test counselling uptake and linkage to anti-retrovirals, where available, were also extracted.

### Results:

Ten eligible studies were identified. Pre-intervention testing uptake ranged from 5.5% to 78.7%. Following intervention A introduction, testing uptake increased by a range of 9.9% to 65.6%, with testing uptake â‰¥ 85% in eight studies. Where reported, pre-test information was provided to between 91.5% and 100% and post-test counselling to between 82% and 99.8% of pregnant women. Linkage to ARVs for prevention of mother to child transmission (PMTCT) was reported in five studies and ranged from 53.7% to 77.2%. Where reported, intervention A was considered acceptable by ANC attendees.

### Limitations:

The review is limited by the variable methodological quality of the included studies and by the significant heterogeneity of interventions and comparison groups. Publication bias cannot be excluded.

### Conclusions:

Our review provides evidence that the adoption of intervention A within ANC can facilitate progress towards universal voluntary testing of pregnant women. This is necessary to increase the coverage of PMTCT services and facilitate access to treatment and prevention interventions. We found some evidence that intervention A adoption does not undermine processes inherent to good conduct of testing, with high levels of pre-test information and post-test counselling, and two studies suggesting that intervention A is acceptable to ANC attendees.

## Abstract 12

### Titre:

Comparator B or intervention A for the management of primary sacrococcygeal pilonidal disease? A meta-analysis of randomized controlled trials.

### Aim:

Sacrococcygeal pilonidal disease is a common condition afflicting the young male working and student population, resulting in considerable pain, embarrassment and loss of workdays. Controversy surrounds the most appropriate surgical approach to achieve low recurrence rates whilst minimizing morbidity and permitting an early return to work. This study aims to review the published literature comparing excision followed by comparator B or intervention A.

### Methods:

PubMed, EMBASE, MEDLINE and The Cochrane Library were systematically reviewed, by two independent investigators, for relevant randomized controlled trials. Keywords and MeSH terms included 'pilonidal disease', 'intervention B/repair', 'intervention A' and 'limberg/modified Limberg flap'. 'Related study' function and manuscript bibliographies were searched for further relevant studies. Study quality was assessed using the Jadad score. Meta-analysis was performed on pooled data, utilizing a random effects model when heterogeneity was high and a fixed effects model when heterogeneity was low. The primary end-point assessed was disease recurrence. Secondary end-points included wound dehiscence, pain scores, hospital stay and return to work.

### Results:

Six studies were eventually included for pooled analysis following exclusion of randomized controlled trials with poor methodology. Two studies compared comparator B with the intervention A. Six hundred and forty-one patients were included (331 intervention B). Intervention A demonstrated a trend towards less disease recurrence (P = 0.07), lower wound infection (P = 0.001) and dehiscence (P = 0.01). However, no significant difference was found for pain scores, hospital stay or return to work.

### Limitations:

The review is limited by the small sample sizes of the included studies and by short term follow up data.

### Conclusions:

The current published literature supports the use of the intervention A procedures over comparator B techniques for the elective management of primary pilonidal disease. Further high-quality studies are necessary to compare intervention A and B.

## Abstract 13

### Titre:

A systematic review and meta-analysis of the effect of intervention A vs. intervention B on visceral fat.

### Abstract:

It is increasingly recognized that the location of excess adiposity, particularly increased deposition of visceral adipose tissue (VAT), is important when determining the adverse health effects of overweight and obesity. Exercise therapy is an integral component of obesity management, but the most potent exercise prescription for VAT benefit is unclear. We aimed to evaluate the independent and synergistic effects of intervention A and intervention B and to directly compare the efficacy of intervention A and intervention B for beneficial VAT modulation. A systematic review and meta-analysis was performed to assess the efficacy of exercise interventions on VAT content/volume in overweight and obese adults. Relevant databases were searched to November 2010. Included studies were randomized controlled designs in which intervention A or intervention B in isolation or combination were employed for 4 weeks or more in adult humans, where computed tomography (CT) or magnetic resonance imaging (MRI) was used for quantification of VAT pre- and post-intervention. Of the 12196 studies from the initial search, 35 were included. After removal of outliers, there was a significant pooled effect size (ES) for the comparison between intervention A and control (-0.33, 95% CI: -0.52 to -0.14; P < 0.01) but not for the comparison between intervention B and control (0.09, 95% CI: -0.17 to -0.36; P = 0.49). Of the available nine studies, which directly compared intervention A with intervention B, the pooled ES did not reach statistical significance (ES = 0.23, 95% CI: -0.02 to 0.50; P = 0.07 favouring AEx). The pooled ES did not reach statistical significance for interventions that combined intervention A and intervention B vs. control (-0.28, 95% CI: -0.69 to 0.14; P = 0.19), for which only seven studies were available. This review is limited by the methodological quality of the included studies, and by significant heterogeneity across studies. These data suggest that intervention A is central for exercise programmes aimed at reducing VAT, and that intervention A below current recommendations for overweight/obesity management may be sufficient for beneficial VAT modification. Further investigation is needed regarding the efficacy and feasibility of multi-modal training as a means of reducing VAT.

## Abstract 14

### Titre:

Intervention A for treatment of type 2 diabetes mellitus in the clinical setting: systematic review and meta-analysis.

### Objectives:

To assess the efficacy and safety of intervention A compared with metformin as monotherapy, or with other commonly used hypoglycaemic drugs combined with metformin, in adults with type 2 diabetes mellitus.

### Design:

Systematic review and meta-analysis of randomised controlled trials.

### Data sources:

Medline, Embase, the Cochrane Library, conference proceedings, trial registers, and drug manufacturers' websites.

### Eligibily Criteria

Randomised controlled trials of adults with type 2 diabetes mellitus that compared intervention A with metformin as monotherapy or with a sulfonylurea, pioglitazone, a glucagon-like peptide-1 (GLP-1) agonist, or basal insulin combined with metformin on the change from baseline in glycated haemoglobin (HbA(1c)).

Data extraction

The primary outcome was the change in HbA(1c). Secondary outcomes included the proportion of patients achieving the goal of HbA(1c) <7%, the change in body weight, discontinuation rate because of any adverse event, occurrence of any serious adverse event, all cause mortality, and incidence of hypoglycaemia, nasopharyngitis, urinary tract infection, upper respiratory infection, nausea, vomiting, and diarrhoea.

### Results:

27 reports of 19 studies including 7136 patients randomised to intervention A and 6745 patients randomised to another hypoglycaemic drug were eligible for the systematic review and meta-analysis. Compared with metformin as monotherapy, intervention A were associated with a smaller decline in HbA(1c) (weighted mean difference 0.20, 95% confidence interval 0.08 to 0.32) and in body weight (1.5, 0.9 to 2.11). As a second line treatment, intervention A were inferior to GLP-1 agonists (0.49, 0.31 to 0.67) and similar to pioglitazone (0.09, -0.07 to 0.24) in reducing HbA(1c) and had no advantage over sulfonylureas in the attainment of the HbA(1c) goal (risk ratio in favour of sulfonylureas 1.06, 0.98 to 1.14). Intervention A had a favourable weight profile compared with sulfonylureas (weighted mean difference -1.92, -2.34 to -1.49) or pioglitazone (-2.96, -4.13 to -1.78), but not compared with GLP-1 agonists (1.56, 0.94 to 2.18). Only a minimal number of hypoglycaemias were observed in any treatment arm in trials comparing intervention A with metformin as monotherapy or with pioglitazone or a GLP-1 agonist as second line treatment. In most trials comparing intervention A with sulfonylureas combined with metformin, the risk for hypoglycaemia was higher in the group treated with a sulfonylurea. Incidence of any serious adverse event was lower with intervention A than with pioglitazone. Incidence of nausea, diarrhoea, and vomiting was higher in patients receiving metformin or a GLP-1 agonist than in those receiving intervention A. Risk for nasopharyngitis, upper respiratory tract infection, or urinary tract infection did not differ between intervention A and any of the active comparators.

### Limitations:

This review is limited by the methodological quality of the included studies. Overall risk of bias for the primary outcome was low in three studies, unclear in nine, and high in 14.

### Conclusions:

In patients with type 2 diabetes who do not achieve the glycaemic targets with metformin alone, intervention A can lower HbA (1c), in a similar way to sulfonylureas or pioglitazone, with neutral effects on body weight. Increased unit cost, which largely exceeds that of the older drugs, and uncertainty about their long term safety, however, should also be considered.

## Abstract 15

### Titre:

Intervention A in heavy menstrual bleeding: a benefit-risk review.

### Abstract:

Heavy menstrual bleeding (HMB) is a common problem in women of reproductive age and can cause irritation, inconvenience, self-consciousness and fear of social embarrassment. Our objective was to review and appraise literature identified from the MEDLINE and EMBASE databases to evaluate the clinical evidence and provide an update on the risks and benefits of using the intervention A in the treatment of HMB. The intervention A consistently reduces menstrual blood loss (MBL) in women with HMB, including those with underlying uterine pathology or bleeding disorders. The available data suggest that it reduces MBL to a greater extent than other medical therapies, including combined oral contraceptives, oral progestogens (both short- or long-cycle regimens), tranexamic acid and oral mefenamic acid. In addition, the intervention A and endometrial ablation appear to reduce MBL to a similar extent. The adverse effects reported with the intervention A in women with HMB are similar to those typically observed in women using the system for contraception. Uterine perforations were not reported in any of the studies reviewed, but expulsion rates may be higher than in the general population of intervention A users. Overall, the intervention A has a positive effect on most quality-of-life domains, at least comparable to those achieved with hysterectomy or endometrial ablation, and is consistently a cost-effective option across a variety of countries and settings. This review is limited by the lack of information to assess the quality of the included studies and by a lack of long term data. Further, publication bias cannot be excluded. In conclusion, the intervention A is an effective treatment option for women with HMB, including those with underlying organic pathology or bleeding disorders.

## Abstract 16

### Titre:

Effects of intervention A on mortality and hospitalization. A meta-analysis of randomized controlled studies.

### Objectives:

Catecholaminergic inotropes have a place in the management of low output syndrome and decompensated heart failure but their effect on mortality is debated. Intervention A is a calcium sensitizer that enhances myocardial contractility without increasing myocardial oxygen use. A meta-analysis was conducted to determine the impact of intervention A on mortality and hospital stay.

### Data sources:

BioMedCentral, PubMed, Embase, and the Cochrane Central Register of clinical trials were searched for pertinent studies. International experts and the manufacturer were contacted.

### Study selection:

Articles were assessed by four trained investigators, with divergences resolved by consensus. Inclusion criteria were random allocation to treatment and comparison of intervention A vs. control. There were no restrictions on dose or time of intervention A administration or on language. Exclusion criteria were: duplicate publications, nonadult studies, oral administration of intervention A, and no data on main outcomes.

### Data extraction:

Study end points, main outcomes, study design, population, clinical setting, intervention A dosage, and treatment duration were extracted.

### Data synthesis:

Data from 5,480 patients in 45 randomized clinical trials were analyzed. The overall mortality rate was 17.4% (507 of 2,915) among intervention A-treated patients and 23.3% (598 of 2,565) in the control group (risk ratio 0.80 [0.72; 0.89], p for effect <.001, number needed to treat = 17 with 45 studies included). Reduction in mortality was confirmed in studies with placebo (risk ratio 0.82 [0.69; 0.97], p = .02) or dobutamine (risk ratio 0.68 [0.52-0.88]; p = .003) as comparator and in studies performed in cardiac surgery (risk ratio 0.52 [0.35; 0.76] p = .001) or cardiology (risk ratio 0.75 [0.63; 0.91], p = .003) settings. Length of hospital stay was reduced in the intervention A group (weighted mean difference = -1.31 [-1.95; -0.31], p for effect = .007, with 17 studies included). A trend toward a higher percentage of patients experiencing hypotension was noted in intervention A vs. control (risk ratio 1.39 [0.97-1.94], p = .053).

### Limitations:

This review is limited by the variable methodological quality of the included studies and by the clinical heterogeneity (setting, dose, control treatment and follow up duration) across studies.

### Conclusions:

Intervention A might reduce mortality in cardiac surgery and cardiology settings of adult patients.

## Abstract 17: vignette 50, avec section limite

### Titre:

Does intervention A versus usual intervention prevent stroke?

### Objectives:

The purpose of this study was to compare the impact of intervention A versus usual intervention on stroke prevention through meta-analysis of randomized controlled trials.

### Methods:

We searched PubMed and bibliographies of recent review articles. Relative risk with 95% confidence interval was used as a measure of the association between intervention A group versus a comparator group, and risk of stroke after pooling data across trials.

### Methods:

The search identified 11 studies with 42,572 participants and 794 stroke events. The final SBPs, weighted for trial size, were a mean of 126.5 mmHg in the intervention A arms and 132.6 mmHg in the conventional arms (mean SBP reduction, 6.1 mmHg). Intervention A was associated with a lower stroke risk (relative risk, 0.80; 95% confidence interval, 0.70-0.92; p < 0.01) and a lower risk of major vascular events (0.91; 0.86-0.96; p < 0.001). In subgroup analyses, subjects with risk factors but no established cardiovascular disease showed substantial reduction of future stroke risk with intervention A (0.49; 0.34-0.69), but those with established cardiovascular disease at entry did not experience stroke risk reduction with intervention A (0.92; 0.83-1.03).

### Limitations:

This review is limited by the risk of publications bias with underrepresentation of small studies showing neutral or adverse effects on the funnel plot.

### Interpretation:

Intervention A compared to usual intervention appears to provide additional stroke protection only among people with risk factors but no established cardiovascular disease.

## Abstract 18

### Titre:

Intervention A versus comparator B for temporomandibular disorders: a systematic review and meta-analysis.

### Purpose:

To compare the effect and safety of intervention A versus comparator B in the treatment of temporomandibular disorders.

### Materials methods:

MEDLINE (via Ovid, 1948 to March 2011), CENTRAL (Issue 1, 2011), Embase (1984 to March 2011), CBM (1978 to March 2011), and World Health Organization International Clinical Trials Registry Platform were searched electronically; relevant journals as well as references of included studies were hand-searched for randomized controlled trials comparing effect or safety of intervention A with comparator B. Risk of bias assessment with the tool recommended by Cochrane Collaboration, reporting quality assessment with CONSORT and data extraction, were carried out independently by 2 reviewers. Meta-analysis was delivered with RevMan 5.0.23.

### Results:

Four trials with 349 participants were included. Meta-analysis showed that intervention A could significantly increase 2.88 mm more maximal mouth opening (P = .0001) and alleviate pain intensity in the temporomandibular area on average by 9.01 mm visual analog scale scores (P = .0001) compared with comparator B, but could not markedly change synthesized clinical index (P = .05) in the short term; nevertheless, they showed more beneficial maximal mouth opening (P = .002), pain relief (P < .0001), and synthesized clinical variable (P < .0001) in the long term than comparator B. No serious adverse events were reported.

### Limitations:

This review is limited by the risk of bias of the included studies and the small sample size and clinical heterogeneity across studies.

### Conclusions:

Intervention A shows better effect than comparator B, and their safety is affirmative. However, more high-quality studies are still needed to test and verify the evidence.

## Abstract 19

### Titre:

Clinical outcomes of comparator B and intervention A for small hepatocellular carcinoma: a meta-analysis.

### Background aim:

To evaluate the evidence comparing comparator B and intervention A on the treatment of hepatocellular carcinoma (HCC) using meta-analytical techniques.

### Methods:

Literature search was undertaken until March 2011 to identify comparative studies evaluating survival rates, recurrence rates, and complications. Pooled odds ratios (OR) and 95% confidence intervals (95% CI) were calculated with either the fixed or random effect model.

### Results:

These studies included a total of 877 patients: 441 treated with comparator B and 436 treated with intervention A. The overall survival was significantly higher in patients treated with intervention A than comparator B at 1, 3 and 5 years (respectively: OR: 0.50, 95% CI: 0.29-0.86; OR: 0.51, 95% CI: 0.28-0.94; OR: 0.62, 95% CI: 0.45-0.84). In the intervention A group the 1, 3, and 5 years recurrence-free survival rates were significantly higher than the comparator B group (respectively: OR: 0.65, 95% CI: 0.44-0.97; OR: 0.65, 95% CI: 0.47-0.89; OR: 0.52, 95% CI: 0.35-0.77). Comparator B had a higher rate of local recurrence (OR: 4.08, 95% CI: 2.03-8.20). For tumors â‰¤ 3 cm intervention A was better than comparator B in the 3-year overall survival rates (OR: 0.38, 95% CI: 0.16-0.89).

### Limitations:

This review is limited by the poor methodological quality and small sample size of the included studies. Asymmetry of the funnel plot suggests possible publication bias.

### Conclusions:

Intervention A was superior to comparator B in the treatment of HCC. However, the findings have to be carefully interpreted due to the lower level of evidence.

## Abstract 20

### Titre:

The efficacy and safety of intervention A for the irritable bowel syndrome: a systematic review and meta-analysis.

### Objectives:

Irritable bowel syndrome (IBS) affects 10-15% of the population, and treatment options are limited. Intervention A is a minimally absorbed antibiotic that has shown efficacy in IBS patients. The objective of our study was to perform a meta-analysis and systematic review of available randomized, placebo controlled trials evaluating the efficacy and tolerability of intervention A in patients with IBS.

### Methods:

We performed a systematic literature search of multiple online electronic databases regardless of language. Inclusion criteria entailed randomized, placebo controlled trials and IBS defined by accepted symptom-based criteria. Meta-analysis was conducted to evaluate the summary odds ratios (ORs) and 95% confidence intervals (CIs) of combined studies for the primary and secondary outcomes using a random-effects model based on the DerSimonian and Laird method to reflect both within- and between study variability. We assessed heterogeneity using X(2) test and the inconsistency index statistic (I(2)). Significant heterogeneity was defined as I(2) â‰¥ 25%. Meta-regression was performed using generalized linear mixed-effects model and study as random effects to estimate the summary OR adjusting for covariate differences across studies and treatment group. Publication bias was assessed by funnel plot analysis.

### Results:

Systematic review identified 13,700 citations. Eighteen were deemed to be potentially relevant, of which five articles met eligibility. Meta-analysis found intervention A to be more efficacious than placebo for global IBS symptom improvement (OR=1.57; 95% CI=1.22, 2.01; therapeutic gain=9.8%; number needed to treat (NNT)=10.2), with mild heterogeneity (P=0.25, I(2)=26%). For the key secondary outcome of bloating, raw data were available for four studies. Intervention A was significantly more likely to improve bloating than placebo (OR=1.55; 95% CI=1.23-1.96; therapeutic gain=9.9%; NNT=10.1), with no significant heterogeneity (P=0.27, I(2)=23%). We found that studies with older patients and more females demonstrated higher response rates, which was consistent regardless of treatment group. In addition, studies with higher cumulative dose tended to report a higher response rate. Of the covariates evaluated, we found age to be most predictive of response, with a correlation coefficient of 0.97 between aggregate response rate and mean age in the placebo groups. Although studies with higher cumulative dose tended to show increased response rates, this was also seen consistently in both the treated and placebo groups. Adverse effects were similar among patients receiving intervention A or placebo in all studies. The most common adverse events (AEs) (â‰¤10%) with intervention A were headache, upper respiratory infection, nausea, nasopharygitis, diarrhea, and abdominal pain. Serious AEs were rare (<1%) and similar with intervention A and placebo.

### Limitations:

The review is limited by the small number of the included studies, evidence of selective reporting of outcome in one study and the risk of publication bias.

### Conclusions:

Intervention A proved more effective than placebo for global symptoms and bloating in IBS patients. The modest therapeutic gain was similar to that yielded by other currently available therapies for IBS. AEs were similar between intervention A and placebo.

## Abstract 21

### Titre:

Does intervention A change physical activity in people with COPD? A systematic review and meta-analysis.

### Abstract:

A systematic review and meta-analysis was conducted to examine the effect of intervention A on daily physical activity (PA) in people with chronic obstructive pulmonary disease (COPD). MEDLINE, PubMed, EMBASE, CINAHL, Physiotherapy Evidence Database (PEDro) and Cochrane Central Register of Controlled Trials were searched from their inception to week 27 of 2010, using the keywords 'COPD,' ' intervention A,' 'therapy' and 'physical activity.' All studies except case reports were eligible for inclusion provided they investigated the effects of â‰¥ 4 weeks of supervised intervention A on PA in patients with COPD. Study quality for the randomised trials (RTs) and single-group interventional studies was rated using the PEDro scale and Downs and Black Tool, respectively. No randomised controlled trials met our study criteria. The two RTs had a mean PEDro score of 5. The 5 single-group studies had a mean Downs and Black score of 19 Â± 3. When combined, a small effect on PA outcomes was demonstrated (overall mean effect = 0.12; p = 0.01). This review is limited by the poor methodological quality of the included studies. The asymmetry of the funnel plot suggests possible publication bias. Taken together, the RTs and single-group studies demonstrate that intervention A may confer a significant but small increase in PA.

## Abstract 22

### Titre:

The efficacy of intervention A for reducing healthcare-associated bloodstream infections: a meta-analysis.

### Design:

Systematic review and meta-analysis of randomized controlled trials and quasi-experimental studies to assess the efficacy of intervention A for prevention of healthcare-associated bloodstream infections (BSIs).

### Setting:

Medical, surgical, trauma, and combined medical-surgical intensive care units (ICUs) and long-term acute care hospitals.

### Participants:

Inpatients.

### Methods:

Data on patient population, diagnostic criteria for BSIs, form and concentration intervention A, incidence of BSIs, and study design were extracted.

### Results:

One randomized controlled trial and 11 nonrandomized controlled trials reporting a total of 137,392 patient-days met the inclusion criteria; 291 patients in the intervention A arm developed a BSI over 67,775 patient-days, compared with 557 patients in the control arm over 69,617 catheter-days. Intervention A resulted in a reduced incidence of BSIs: the pooled odds ratio using a random-effects model was 0.44 (95% confidence interval, 0.33-0.59; [Formula: see text]). Statistical heterogeneity was moderate, with an I(2) of 58%. For the subgroup of studies that examined central line-associated BSIs, the odds ratio was 0.40 (95% confidence interval, 0.27-0.59).

### Limitations:

This review is limited by poor methodological quality of the included studies, lack of assessement of potential confounding and high clinical heterogeneity.

### Conclusions:

Intervention A reduced the incidence of BSIs, including central line-associated BSIs, among patients in the medical ICU. Further studies are recommended to determine the optimal frequency, method of application, and concentration intervention A as well as the comparative effectiveness of this strategy relative to other preventive measures available for reducing BSIs. Future studies should also examine the efficacy of intervention A in non-ICU populations at risk for BSI.

## Abstract 23

### Titre:

Intervention A in women with an asymptomatic sonographic short cervix in the midtrimester decreases preterm delivery and neonatal morbidity: a systematic review and metaanalysis of individual patient data.

### Objectives:

To determine whether the use of Intervention A in asymptomatic women with a sonographic short cervix (â‰¤ 25 mm) in the midtrimester reduces the risk of preterm birth and improves neonatal morbidity and mortality.

### Study design:

Individual patient data metaanalysis of randomized controlled trials.

### Results:

Five trials of high quality were included with a total of 775 women and 827 infants. Treatment with intervention A was associated with a significant reduction in the rate of preterm birth <33 weeks (relative risk [RR], 0.58; 95% confidence interval [CI], 0.42-0.80), <35 weeks (RR, 0.69; 95% CI, 0.55-0.88), and <28 weeks (RR, 0.50; 95% CI, 0.30-0.81); respiratory distress syndrome (RR, 0.48; 95% CI, 0.30-0.76); composite neonatal morbidity and mortality (RR, 0.57; 95% CI, 0.40-0.81); birthweight <1500 g (RR, 0.55; 95% CI, 0.38-0.80); admission to neonatal intensive care unit (RR, 0.75; 95% CI, 0.59-0.94); and requirement for mechanical ventilation (RR, 0.66; 95% CI, 0.44-0.98). There were no significant differences between the intervention A and placebo groups in the rate of adverse maternal events or congenital anomalies.

### Limitations:

This review is limited by the small number and small sample size of the included studies. Publication bias cannot be excluded.

### Conclusions:

Intervention A administration to asymptomatic women with a sonographic short cervix reduces the risk of preterm birth and neonatal morbidity and mortality.

## Abstract 24

### Titre:

Intervention A to protect young children: a systematic review and meta-analysis.

### Background:

Young children can be protected from much of the harm from tobacco smoke exposure if their parents quit smoking. Some researchers encourage parents to quit for their children's benefit, but the evidence for effectiveness of such approaches is mixed.

### Objectives:

To perform a systematic review and meta-analysis to quantify the effects of intervention A.

### Methods:

We searched PubMed, the Cochrane Library, Web of Science, and PsycINFO. Controlled trials published before April 2011 that targeted smoking parents of infants or young children, encouraged the intervention A for their children's benefit, and measured of intervention A results rates were included. Study quality was assessed. Relative risks and risk differences were calculated by using the DerSimonian and Laird random-effects model.

### Results:

Eighteen trials were included. Interventions took place in hospitals, pediatric clinical settings, well-baby clinics, and family homes. Intervention A success rates averaged 23.1% in the intervention group and 18.4% in the control group. The interventions A successfully increased the parental quit rate. Subgroups with significant intervention benefits were children aged 4 to 17 years, interventions whose primary goal was cessation, interventions that offered medications, and interventions with high follow-up rates (>80%).

### Limitations:

This review is limited by the clinical heterogeneity across included studies and by the short-term outcome for several studies. Also the methodological quality of the included studies is variable from high to moderate quality.

### Conclusions:

Intervention A, for the sake of the children, provide a worthwhile addition to the arsenal of cessation approaches, and can help protect vulnerable children from harm due to tobacco smoke exposure. However, most parents do not quit, and additional strategies to protect children are needed.

## Abstract 25

### Titre:

Prophylaxis of radiation-induced nausea and vomiting using intervention A: a systematic review of randomized trials.

### Purpose:

To systematically review the effectiveness and safety of intervention A compared with other antiemetic medication or placebo for prophylaxis of radiation-induced nausea and vomiting.

### Materials methods:

We searched the following electronic databases: MEDLINE, Embase, the Cochrane Central Register of Controlled Clinical Trials, and Web of Science. We also hand-searched reference lists of included studies. Randomized, controlled trials that compared intervention A with another antiemetic medication or placebo for preventing radiation-induced nausea and vomiting were included. We excluded studies recruiting patients receiving concomitant chemotherapy. When appropriate, meta-analysis was conducted using Review Manager (v5) software. Relative risks were calculated using inverse variance as the statistical method under a random-effects model. We assessed the quality of evidence by outcome using the Grading of Recommendations Assessment, Development, and Evaluation approach.

### Results:

Eligibility screening of 47 articles resulted in 9 included in the review. Meta-analysis of intervention A vs. placebo showed significant benefit for intervention A (relative risk [RR] 0.70; 95% confidence interval [CI] 0.57-0.86 for emesis; RR 0.84, 95% CI 0.73-0.96 for nausea). Meta-analysis comparing intervention A vs. metoclopramide showed a significant benefit of the intervention A for emetic control (RR 0.27, 95% CI 0.15-0.47).

### Limitations:

This review is limited by the poor methodological quality of the included studies, and by the small sample size. Publication bias cannot be excluded.

### Conclusions:

Interventions A are superior to placebo and other antiemetics for prevention of emesis, but little benefit was identified for nausea prevention. Interventions A are suggested for prevention of emesis. Limited evidence was found regarding delayed emesis, adverse events, quality of life, or need for rescue medication. Future randomized, controlled trials should evaluate interventions A and new agents with novel mechanisms of action such at the NK(1) receptor antagonists to determine the most effective drug. Delayed nausea and vomiting should be a focus of future study, perhaps concentrating on the palliative cancer population.

## Abstract 26

### Titre:

Efficacy and safety of intervention A versus comparator B during percutaneous coronary intervention: systematic review and meta-analysis.

### Objectives:

To determine the efficacy and safety of intervention A compared with comparator B during percutaneous coronary intervention.

### Design:

Systematic review and meta-analysis.

### Data sources:

Medline and Cochrane database of systematic reviews, January 1996 to May 2011.

### Study selection:

Randomised and non-randomised studies comparing intervention A with comparator B during percutaneous coronary intervention and reporting on both mortality (efficacy end point) and major bleeding (safety end point) outcomes.

### Data extraction:

Sample size, characteristics, and outcomes, extracted independently and analysed.

### Data synthesis:

23 trials representing 30,966 patients were identified, including 10,243 patients (33.1%) undergoing primary percutaneous coronary intervention for ST elevation myocardial infarction, 8750 (28.2%) undergoing secondary percutaneous coronary intervention after fibrinolysis, and 11,973 (38.7%) with non-ST elevation acute coronary syndrome or stable patients scheduled for percutaneous coronary intervention. A total of 13,943 patients (45.0%) received intervention A and 17,023 (55.0%) comparator B. Intervention A was associated with significant reductions in death (relative risk 0.66, 95% confidence interval 0.57 to 0.76; P<0.001), the composite of death or myocardial infarction (0.68, 0.57 to 0.81; P<0.001), and complications of myocardial infarction (0.75, 0.6 to 0.85; P<0.001), and a reduction in incidence of major bleeding (0.80, 0.68 to 0.95; P=0.009). In patients who underwent primary percutaneous coronary intervention, the reduction in death (0.52, 0.42 to 0.64; P<0.001) was particularly significant and associated with a reduction in major bleeding (0.72, 0.56 to 0.93; P=0.01).

### Limitations:

This review is limited by the methodological quality of the included studies. The meta analysis included 11 RCTs, one prospective study of RCT, seven registry studies and four retrospective analyses of RCTs.

### Conclusions:

Intervention A seems to be superior to comparator B in reducing mortality and bleeding outcomes during percutaneous coronary intervention and particularly in patients undergoing primary percutaneous coronary intervention for ST elevation myocardial infarction.

## Abstract 27

### Titre:

The effect of intervention A on work participation in rheumatoid arthritis patients: a systematic review.

### Abstract:

This study reviewed the effect of intervention A on participation in paid work among patients with rheumatoid arthritis (RA). A systematic literature search was performed to identify published articles reporting the effect of intervention A on employment status, sick leave and/or presenteeism. The quality of included articles was assessed according to the guidelines as proposed by the Dutch Cochrane Centre. Narrative summaries were used to present the data separately for randomised controlled trials (RCTs) as well as controlled and uncontrolled cohort studies. 19 studies (six uncontrolled cohorts, seven controlled cohorts and six RCTs) were included, in which 11 259 patients were treated with intervention A . Employment status improved in four out of 13 studies, absence from work in all 10 studies and presenteeism in seven out of nine studies that reported this outcome. For absenteeism and presenteeism the statistical significance of change or difference was not always provided and results within studies were sometimes conflicting when using different time frames or alternative outcomes. This study is limited by the methodological quality of the included studies, and significant heterogeneity in terms of population, design, analyses and most important in outcome measures. RCTs as well as cohort studies showed positive results of intervention A on both absenteeism and presenteeism compared with other disease-modifying antirheumatic drugs (DMARD), continuing the failing DMARD, the general population or the situation before the start of intervention A. The effect on employment status was more conflicting, but 50% of studies that addressed patients with early methotrexate-naive RA showed a positive result on employment status.

## Abstract 28

### Titre:

Interventions A for the treatment of non-alcoholic fatty liver disease in adults: a systematic review.

### Abstract:

Non-alcoholic fatty liver disease is a serious and growing clinical problem. Despite intervention A being the recommended therapy, there are currently no systematic evaluations of its efficacy. This review applies a systematic approach to evaluating intervention A studied to date. Medline (Pubmed), Scopus, and the Cochrane Controlled Trials Register were searched for studies and study groups assessing the effect of intervention A in adult populations with non-alcoholic fatty liver disease. The outcome markers of interest were indicators of steatosis, histological evidence of inflammation and fibrosis, and glucose control/insulin sensitivity. We identified 23 studies for inclusion; seven had control groups, but only six were randomised. Eleven groups received intervention A type 1, two intervention A type 2, and 19 intervention A type 1 and 2. Studies consistently showed reductions in liver fat and/or liver aminotransferase concentration, with the strongest correlation being with weight reduction. Of the 5 studies reporting changes in histopathology, all showed a trend towards reduction in inflammation, in 2 this was statistically significant. Changes in fibrosis were less consistent with only one study showing a significant reduction. The majority of studies also reported improvements in glucose control/insulin sensitivity following intervention. This review is limited by the considerable heterogeneity of study design, definition of disease, assessment methods, and interventions across studies and by the poor methodological quality of the included studies. Intervention A leading to weight reduction and/or increased physical activity consistently reduced liver fat and improved glucose control/insulin sensitivity. Limited data also suggest that intervention A may hold benefits for histopathology.

## Abstract 29

### Titre:

Reno-protective effects of interventions A in type 2 diabetic patients: a systematic review and network meta-analysis.

### Aims hypothesis:

This meta-analysis aimed to compare the renal outcomes between intervention A and other antihypertensive drugs or placebo in type 2 diabetes.

### Methods:

Publications were identified from Medline and Embase up to July 2011. Only randomised controlled trials comparing intervention A monotherapy with other active drugs or placebo were eligible. The outcome of end-stage renal disease, doubling of serum creatinine, microvascular complications, microalbuminuria, macroalbuminuria and albuminuria regression were extracted. Risk ratios were pooled using a random-effects model if heterogeneity was present; a fixed-effects model was used in the absence of heterogeneity.

### Results:

Of 673 studies identified, 28 were eligible (n = 13-4,912). In direct meta-analysis, interventions A had significantly lower risk of serum creatinine doubling (pooled RR = 0.66 [95% CI 0.52, 0.83]), macroalbuminuria (pooled RR = 0.70 [95% CI 0.50, 1.00]) and albuminuria regression (pooled RR 1.16 [95% CI 1.00, 1.39]) than other antihypertensive drugs, mainly calcium channel blockers (CCBs). Although the risks of end-stage renal disease and microalbuminuria were lower in the intervention group (pooled RR 0.82 [95% CI 0.64, 1.05] and 0.84 [95% CI 0.61, 1.15], respectively), the differences were not statistically significant. The intervention benefit over placebo was significant for all outcomes except microalbuminuria. A network meta-analysis detected significant treatment effects across all outcomes for both active drugs and placebo comparisons.

### Limitations:

This review is limited by evidence of publication bias and by the methodological quality of the included studies. Also, despite a large overall number, some comparator groups still have small numbers, leading to poor precision of estimates.

### Conclusions interpretation:

Our review suggests a consistent reno-protective effect of intervention A over other antihypertensive drugs, mainly CCBs, and placebo in type 2 diabetes. The lack of any differences in BP decrease between intervention A and active comparators suggest this benefit is not due simply to the antihypertensive effect.

## Abstract 30

### Titre:

Effects of intervention A on weight loss: systematic review and meta-analyses of randomised controlled trials.

### Objectives:

To determine whether treatment with interventions A result in weight loss in overweight or obese patients with or without type 2 diabetes mellitus.

### Design:

Systematic review with meta-analyses.

### Data sources:

Electronic searches (Cochrane Library, Medline, Embase, and Web of Science) and manual searches (up to May 2011).

### Review methods:

Randomised controlled trials of adult participants with a body mass index of 25 or higher; with or without type 2 diabetes mellitus; and who received intervention A type 1 twice daily, intervention A type 1 once weekly, or intervention A type 2 once daily at clinically relevant doses for at least 20 weeks. Control interventions assessed were placebo, oral antidiabetic drugs, or insulin.

### Data extraction:

Three authors independently extracted data. We used random effects models for the primary meta-analyses. We also did subgroup, sensitivity, regression, and sequential analyses to evaluate sources of intertrial heterogeneity, bias, and the robustness of results after adjusting for multiple testing and random errors.

### Results:

25 trials were included in the analysis. Interventions A groups achieved a greater weight loss than control groups (weighted mean difference -2.9 kg, 95% confidence interval -3.6 to -2.2; 21 trials, 6411 participants). The results were confirmed in sequential analyses. We recorded weight loss in the interventions A groups for patients without diabetes (-3.2 kg, -4.3 to -2.1; three trials) as well as patients with diabetes (-2.8 kg, -3.4 to -2.3; 18 trials). In the overall analysis, interventions A had beneficial effects on systolic and diastolic blood pressure, plasma concentrations of cholesterol, and glycaemic control, but did not have a significant effect on plasma concentrations of liver enzymes. Intervention A were associated with nausea, diarrhoea, and vomiting, but not with hypoglycaemia.

### Limitations:

This review is limited by evidence of intertrial heterogeneity. All included trials received industry funding.

### Conclusions:

The present review provides evidence that treatment with interventions A leads to weight loss in overweight or obese patients with or without type 2 diabetes mellitus.

# Invitation e-mail

| Dear colleague, |
| --- |
| We are writing to ask for your help in making medical research more valuable to patients. Given your expertise in the field of clinical research, we would like to invite you to participate in an international academic study to investigate how physicians interpret abstracts. |
| The study has been designed so that it will require only a minimal amount of work on your part and yet provides a real opportunity to actively contribute to improve the communication of research results to inform clinical practice. |
| Your participation would involve in reading 1 abstract of a systematic review and answering 5 short questions about its findings. |
| As a thank for your participation in the study, you will be entered into a draw along with all other participants for a chance to win an **iPad mini**. |
| You can complete the survey by clicking here. |
| Or by copying and pasting the following link into your web browser : "http://www.epidemio-hoteldieu.com/ILMARI2/php/survey.php?num=18&part=1018" |
| With best wishes |
| Dr Amelie Yavchitz (INSERM, France)  Dr Sally Hopewell (University of Oxford, UK)  Pr Philippe Ravaud (Paris Descartes University, INSERM, France)  Pr Isabelle Boutron (Paris Descartes University, INSERM, France) |
| The University of Paris Descartes, Institutional Review Board (IORG0000725), has approved this study. Your responses will be kept confidential.  If you prefer not to receive future reminders regarding this study, please contact Amelie Yavchitz at amelie.yavchitz@bjn.aphp.fr |
|  |
|  |
| © 2013 Centre d 'épidémiologie clinique, Paris |

| Dear colleague, |
| --- |
| A weeks ago we invited you to participate in a study which aims to evaluate the impact of abstract content on readers’ interpretation. |
| We are writing to ask for your help in making medical research more valuable to patients. Given your expertise in the field of clinical research, we would like to invite you to participate in an international academic study to investigate how physicians interpret abstracts. |
| The study has been designed so that it will require only a minimal amount of work on your part and yet provides a real opportunity to actively contribute to improve the communication of research results to inform clinical practice. |
| Your participation would involve in reading 1 abstract of a systematic review and answering 5 short questions about its findings. |
| As a thank for your participation in the study, you will be entered into a draw along with all other participants for a chance to win an **iPad mini**. |
| You can complete the survey by clicking here. |
| Or by copying and pasting the following link into your web browser : "http://www.epidemio-hoteldieu.com/ILMARI2/php/survey.php?num=3845&part=4845" |
| With best wishes |
| Dr Amelie Yavchitz (INSERM, France)  Dr Sally Hopewell (University of Oxford, UK)  Pr Philippe Ravaud (Paris Descartes University, INSERM, France)  Pr Isabelle Boutron (Paris Descartes University, INSERM, France) |
| The University of Paris Descartes, Institutional Review Board (IORG0000725), has approved this study. Your responses will be kept confidential.  If you prefer not to receive future reminders regarding this study, please contact Amelie Yavchitz at amelie.yavchitz@bjn.aphp.fr |
|  |
|  |
| © 2013 Centre d 'épidémiologie clinique, Paris |

# Survey

Based on the information reported in the abstract, please answer the following questions about the intervention.
To avoid any biased interpretation, the description and name of the intervention have been masked.

***Randomized abstract***

1) How confident are you in the results of this study?

0 □ 1 □ 2 □ 3 □ 4 □ 5 □ 6 □ 7 □ 8 □ 9 □ 10 □

« Not confident at all»  « very confident»

2) How confident are you in the validity of the conclusions of this study?

0 □ 1 □ 2 □ 3 □ 4 □ 5 □ 6 □ 7 □ 8 □ 9 □ 10 □

« Not confident at all »  *« very confident»*

3) How confident are you that the intervention “A” could be of benefit to patients?

0 □ 1 □ 2 □ 3 □ 4 □ 5 □ 6 □ 7 □ 8 □ 9 □ 10 □

« Not confident at all»  « very confident»

4) How confident are you that the results of this study could influence your clinical practice?

0 □ 1 □ 2 □ 3 □ 4 □ 5 □ 6 □ 7 □ 8 □ 9 □ 10 □

« Not confident at all »  *« very confident»*

5) Do you think that this systematic review was conducted rigorously?

0 □ 1 □ 2 □ 3 □ 4 □ 5 □ 6 □ 7 □ 8 □ 9 □ 10 □

« not rigorously at all» « yes, very rigorously»

**Page 2**

**Other data collected**

Demographic data

Your age :

Sex : Female Male

Epertise :

Qualification : MD, MD/PhD, Other

How many years have you been a clinician ? <5, 5-10, 10-15, >15

Where are you currently located : France, UK, Other European country, USA, Canada, South america, Asia, Oceania.

Are you working in a ? Primary care center, Secondary care center, Tertiary care center.

How many randomized trial have you been involved in (as principal or co-investigator) ? 1-3, 4-6, 7-10, >10

How many systematic review have you been involved in (as first or co-authors) ? 0, 1-3, 4-6, 7-10, >10

In the last month, approximately how many abstracts published in biomedical journals have you read ? 0, 1-5, 5-10, 10-15, 15-20, >20

In the last year, approximately how many articles have you peer reviewed for a biomedical journal ? 0, 1-5, 5-10, 10-15, 15-20, >20

In the last year, approximately how many grant proposals have you peer reviewed ? 0, 1-5, 5-10, 10-15, 15-20, >20

Did you received some training in Clinical Epidemiology ? Yes No

Did you received some training related to the methods of systematic review ? Yes No

**Page 3**

**Thank you for your participation in our survey**
